# Tracking the neurodegenerative gradient after spinal cord injury

**DOI:** 10.1016/j.nicl.2020.102221

**Published:** 2020-02-25

**Authors:** Michela Azzarito, Maryam Seif, Sreenath Kyathanahally, Armin Curt, Patrick Freund

**Affiliations:** a Spinal Cord Injury Center Balgrist, University Hospital, Zurich, Switzerland; b Department of Neurophysics, Max Planck Institute for Human Cognitive and Brain Sciences, Leipzig, Germany; c Wellcome Trust Centre for Neuroimaging, UCL Institute of Neurology, London, United Kingdom; d Department of Neurology, University Hospital Zurich, Zurich, Switzerland

**Keywords:** Spinal cord injury, MRI, Volumetric MRI, MT

## Abstract

**Objective:**

To quantify neurodegenerative changes along the cervical spinal cord rostral to a spinal cord injury (SCI) by means of quantitative MRI (qMRI) and to determine its relationship with clinical impairment.

**Methods:**

Thirty chronic SCI patients (15 tetraplegics and 15 paraplegics) and 23 healthy controls underwent a high-resolution T1-weighted and myelin-sensitive magnetization transfer (MT) MRI. We assessed macro- and microstructural changes along the cervical cord from levels C1 to C4, calculating cross-sectional spinal cord area, its anterior-posterior and left-right width and myelin content (i.e. MT). Regression analysis determined associations between qMRI parameters and clinical impairment.

**Results:**

In SCI patients, cord area decreased by 2.67 mm^2^ (*p = 0.004*) and left-right width decreased by 0.35 mm (*p = 0.002*) per cervical cord level in the caudal direction when compared to the healthy controls. This gradient of neurodegeneration was greater in tetraplegic than paraplegics in the cross-sectional cervical cord area (by 3.28 mm^2^, *p* = 0.011), left-right width (by 0.36 mm, *p* = 0.03), and mean cord MT (by 0.13%, *p* = 0.04), but independant of lesion severity (*p* > 0.05). Higher lesion level was associated with greater magnitudes of neurodegeneration. Greater loss in myelin content in the dorsal columns and spinothalamic tract was associated with worse light touch (*p* = 0.016) and pin prick score (*p* = 0.024), respectively.

**Conclusions:**

A gradient of neurodegeneration is evident in the cervical cord remote from a SCI. Tract-specific associations with appropriate clinical outcomes highlight that remote neurodegenerative changes are clinically eloquent. Monitoring the neurodegenerative gradient could be used to track treatment effects of regenerative and neuroprotective agents, both in trials targeting cervical and thoracic SCI patients.

## Highlights

• Neurodegenerative gradient along the injured spinal cord after SCI.
• Neurodegenerative gradient predicts level of impairment after SCI.
• Quantitative MRI reveals demyelination as a key contributor to degenertation.

AIS =: American Spinal Injury Association Impairment
APW =: anterior-posterior width
ISNCSCI =: International Standards for Neurological Classification of Spinal Cord Injury
LRW =: left-right width
MPM =: multi-parameter mapping
MT =: magnetization transfer
p.u. =: percentage unit
pSCI =: paraplegics
SC =: spinal cord
SCI =: spinal cord injury
SCIM =: spinal cord independence measure
tSCI =: tetraplegics
VBM =: voxel based morphometry
VBQ =: voxel based quantification

## 1 Introduction

Traumatic spinal cord injury (SCI) leads most often to paralysis below the level of injury and functional recovery is limited (Ahuja et al., 2017). Based on the lesion level, SCI patients can be dichotomized into tetraplegics (tSCI) and paraplegics (pSCI) (Kirshblum et al., 2011). Evidence from experimental SCI have shown that axonal degeneration and demyelination of spinal pathways is greatest close to the injury and decreases with increasing distance (Bresnahan, 1978; Bresnahan et al., 1976; Freund et al., 2007; Grumbles and Thomas, 2017; Kalil and Schneider, 1975; Kerschensteiner et al., 2005). In human SCI, tissue-specific neurodegenerative changes have been quantified in the spinal cord either at the C2/C3 level (Freund et al., 2013, 2011; Huber et al., 2018; Lundell et al., 2011; Seif et al., 2018) or at the lumbar enlargement (David et al., 2019), but not along the cervical spinal cord. Thus it is unknown whether a tract-specific neurodegenerative gradient exists (Bresnahan, 1978; Bresnahan et al., 1976; Kalil and Schneider, 1975; Kerschensteiner et al., 2005) that could be quantified in-vivo. This could for example facilitate monitoring effects of regenerative and neuroprotective treatments. However, a potential gradient of neurodegeneration could be influenced by the level of injury and the anatomical characteristics of the spinal level being affected by a lesion. Therefore, a lesion higher in the cervical cord will injure more axons that project through it (more neurodegeneration) than an identical lesion more caudal in the cord. Moreover, the descending motor tracts (i.e. corticospinal, rubrospinal tracts) located in the lateral funiculi and the ascending sensory pathways (i.e. fasciculus gracilis and cuneatus) located in the dorsal column undergo different types of degeneration. While the descending motor tracts undergo retrograde degeneration, which are greatest in the proximity of the injury (Bresnahan et al., 1976; Kalil and Schneider, 1975), the ascending tracts undergo Wallerian degeneration as their axon stumps are disconnected from their parental neuron.

Here we use the spinal cord toolbox (SCT) (De Leener et al., 2017) to analyse volumetric T1-weigthed MRI data (Tardif et al., 2009) and myelin-sensitive magnetisation transfer saturation (MT) maps (Helms et al., 2008) along the cervical cord from C1 to C4 to assess changes to the cords’ morphometry (Huber et al., 2018) and its myelin content (Schmierer et al., 2004), respectively. Based on the literature (Freund et al., 2019), we hypothesized that (i) a neurodegenerative gradient will be present in SCI patients with more atrophy closer to the injury epicentre (i.e. changes at level C4 > C3 > C2 > C1); (ii) these atrophic changes are more pronounced in tetraplegics when compared to paraplegics given the proximity of this investigated region to the site of injury; (iii) the pathophysiological underpinning of the atrophy is related to demyelination by means of myelin-sensitive MT and; (iv) the magnitude of neurodegeneration is associated with clinical impairment.

## 2 Materials and methods

### 2.1 Participants and study design

Thirty patients with traumatic SCI, 15 tetraplegic (age: mean 42.61 ± SD 17.64 years, 1 female), 15 paraplegic (age: mean 46.73 ± SD 16.09 years, 2 females), and 23 healthy subjects (age = mean 36.87 ± SD 11.76 years, 10 females) were recruited at the University Hospital Balgrist between August 2011 and May 2015. The exclusion criteria for SCI patients were: time since injury <2 months, pregnancy, head or brain lesions associated with spinal cord injury, pre-existing neurological and medical disorders leading to functional impairments, mental disorder, or contraindications to MRI. Note that one of the paraplegics' patients recovered from AIS score D to E during this study (see Table 1). The exclusion criteria for healthy subjects were: any neurological or mental disorder or pregnancy.

**Table 1:** Demographic and clinical information of the spinal cord injury patients

tSCI	Age (years)	Time since injury (months)	sex	AIS	Site of impairment (motor/sensory)	Level of the hyper-intensive signal change	Lower limb motor score	Upper limb motor score	Pinprick	Light touch	SCIM
1	19	13.5	M	A	C6/C7	C5	0	23	33	33	37
2	24	12.2	M	D	T1/C6	C6	19	48	37	72	70
3	43	15.7	M	A	C6/C4	C6	0	25	18	20	37
4	72	11.9	M	D	T1/T2	C7	41	48	41	112	36
5	21	12.3	M	B	C6/C5	C5	0	23	26	53	34
6	31	12.3	M	B	T1/C7	C6	0	48	46	68	38
7	48	12.1	M	D	C1/C1	none	47	35	97	98	98
8	52	9.7	M	C	C7/C5	C6	12	32	44	67	31
9	68	12.1	M	D	C3/C3	C4	50	50	102	107	100
10	34	12.2	M	A	C7/C7	C7	0	35	29	32	26
11	55	18.6	F	D	C3/C3	C4	49	42	94	62	84
12	32	10.3	M	A	C6/C5	C5	0	26	20	33	30
13	29	12.1	M	A	C5/C4	C4	0	14	13	16	19
14	43	186.8	M	B	C6/C4	C4	0	25	32	77	29
15	69	290.5	M	D	T1/C3	C4	40	49	78	69	NA
											
pSCI	Age (years)	Time since injury (months)	sex	AIS	Site of impairment (motor/sensory)	anatomical level of the hyper-intensive signal change	Lower limb motor score	Upper limb motor score	Pinprick	Light touch	SCIM
1	69	12.2	F	D	T11/T11	T6	32	49	74	92	42
2	45	13.4	M	D	L3/L4	none	45	50	106	106	100
3	53	12.0	M	D	T10/T10	T8	48	50	90	90	100
4	30	10.3	M	A	T10/T10	T5	16	50	78	82	80
5	70	9.5	M	A	T7/T7	T8	0	50	68	67	49
6	72	12.0	F	E	intact	T6	50	50	112	112	97
7	53	54.6	M	A	T3/T3	T4	0	50	44	47	53
8	36	185.5	M	A	T12/T12	T11	4	50	78	78	70
9	60	68.2	M	A	T4/T4	T5	0	49	40	52	32
10	53	8.0	M	A	T9/T9	T7	0	50	66	68	69
11	32	10.8	M	B	T11/T11	T11	0	50	72	78	66
12	29	22.8	M	B	T6/T6	T4	0	50	52	77	66
13	26	10.8	M	A	T4/T4	T5	0	50	46	48	67
14	39	9.3	M	A	T7/T7	T8	0	50	58	60	65
15	31	12.3	M	B	T4/T4	T5	0	50	46	74	54

Abbreviations: AIS: American Spinal Injury Association Impairment Scale; F: female; M: male; tSCI: patients with tetraplegia;

pSCI: patients with paraplegia; SCIM: Spinal Cord Independence Measure

### 2.2 Standard protocol approvals, registrations, and patient consents

The study was approved by the local ethics committee of Zurich the ‘Kantonale Ethikkommission Zurich’ (EK-2010-0271), and the study protocols were in accordance with the Declaration of Helsinki. All subjects provided written informed consent prior to enrolment.

### 2.3 Clinical assessments

SCI patients underwent a comprehensive clinical protocol including the International Standards for Neurological Classification of Spinal Cord Injury (ISNCSCI) (Kirshblum et al., 2011) for motor, light touch, and pin prick score; the Spinal Cord Independence Measure (SCIM) (Catz et al., 2007); and the Graded Redefined Assessment of Strength, Sensibility, and Prehension (GRASSP) particularly in tetraplegics patients (Kalsi-Ryan et al., 2012) (Table 1).

### 2.4 Image acquisition

Participants underwent a T1-weighted 3D Magnetization Prepared Rapid Acquisition Gradient-Echo (MPRAGE) scan (whole-brain including the cervical cord down to C4 level) on a 3T MRI scanner (Magnetom Skyra^Fit^ and Verio, Siemens Healthcare, Erlangen, Germany). The system was equipped with a 16-channel radiofrequency (RF) receive head and neck coil and RF body transmit coil. The MRI parameters used in T1-weighted scan were as follows: field of view (FOV) of 224 × 256 mm^2^, matrix size of 224 × 256, isotropic resolution of 1 mm^3^, repetition time (TR) = 2420 ms, echo time (TE) = 4.18 ms, flip angle(α) = 9°, inversion time = 960 ms, and readout bandwith of 150 Hz per pixel.

To assess microstructural changes (myelination) associated with quantitative MR parameters, a whole-brain multi-parameter mapping (MPM) protocol (Callaghan et al., 2015; Helms et al., 2009, 2008; Weiskopf et al., 2013, 2011) was performed using a 3D multi-echo fast low-angle shot (FLASH) gradient-echo sequence. MPM protocol is designed to provide MR parameter measures of longitudinal relaxation rate (R1 = 1/T1), effective proton density (PD*), magnetization transfer saturation (MT) and effective transverse relaxation rate (R2* = 1/T2*). MRI scans cover the whole brain and cervical cord down to the C4 level with 1 mm isotropic resolution, FOV = 240 × 256 mm^2^ and matrix-size = 5240 × 256, with 176 partitions. Total scan time was 23 min applying parallel imaging in the phase-encoding direction using a generalized auto-calibration partially parallel acquisition algorithm (GRAPPA) factor 2 × 2 and readout bandwidth of 480 Hz/pixel. MPM protocol consists of three different MRI weighted contrasts, each acquired using a different TR and flip angle (α):(1) T1-weighted scan: 25 ms / 23°, (2) PD-weighted scan: 25 ms / 4°, and (3) MT-weighted scan: 37 ms / 9° with off-resonance RF pulse prior to excitation. Echoes were acquired at six equidistant times of echo (TE) from 2.46 ms to 17.22 ms for all volumes and an additional echo time at 19.68 ms for PD-weighted and T1-weighted scans.

### 2.5 MRI Data processing

MT-weighted, PD-weighted, and T1-weighted images acquired from MPM protocol were used to compute quantitative maps of MT, R1 and R2* using SPM12 (University College London, London, UK). The quality at cervical level C4 for R1 and R2* maps, after visual inspection, was insufficient and therefore could not be reliably analysed.

**Fig. 1:**
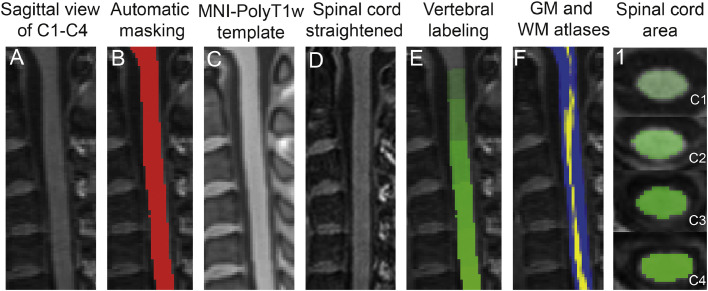
Pre-processing steps for the analysis of MRI scans. Image registration pipeline for T1-weighted MRI from a healthy subject to PAM50 template using the spinal cord toolbox. The original T1-weighted image (A) was segmented using deep segmentation(Perone et al., 2018) and the spinal cord mask was generated (B). Next, the template (MNI-poly T1w) was warped into subject space (C) and the T1-weighted image was warped into template space (straightened) (D). Vertebral levels were automatically labelled (E) and GM (yellow) and WM (blue) probabilistic atlases were warped back to native space (sagittal view in F) (De Leener et al., 2017). Segmented SC from C1 level down to C4 is presented in column 1. (For interpretation of the references to color in this figure legend, the reader is referred to the web version of this article.)

T1-weighted images (MPRAGE) and MT maps were analysed using spinal cord toolbox (SCT) (De Leener et al., 2017) following the automatic pipeline to register, warp, and extract morphometric and microstructural parameters. The pipeline included automatic spinal cord segmentation followed by visual inspection and manual correction of the generated masks if necessary (in FSL; https://fsl.fmrib.ox.ac.uk/). Next, the generated spinal cord mask was registered to the MNI-Poly-AMU template (De Leener et al., 2018) using a combination of affine and nonlinear registrations, and the reverse deformation field (template to subject) was applied to the white matter (WM) and gray matter (GM) atlases, projecting them into the subject space. Finally, morphometric and microstructural parameters were extracted automatically from the spinal cord at each level (from cervical level C1 down to cervical level C4). Morphometric SC parameters consisting of anterior-posterior width (APW), left-right width (LRW) and spinal cord cross-sectional area (SCA) were extracted from the segmented T1-weighted. Myelin sensitive MT values were extracted from the segmented MT map. Fig. 1 demonstrates the processing pipeline. Voxel-wise analysis was performed on the MT maps in SPM12 after warping into template space (PAM50) and applying an anisotropic smoothing along the main axis (gaussian kernel with sigma 3 mm in SCT).

### 2.6 Statistical analysis

To investigate the difference between SCI patients and healthy controls in spinal cord morphometry (i.e. cord area, APW and LRW) and microstructure (i.e. MT), a linear mixed effect model across the cervical segments (from C1 to C4) and across groups (healthy controls, both patients' groups combined, as well as pSCI and tSCI separately) was conducted in Stata (Stata Corp 13.0, College Station, TX). Post-hoc pairwise comparisons were performed, adjusted for multiple comparisons using Bonferroni correction. To assess lesion completeness effects (AIS scores), spinal cord parameters at all spinal cord levels were compared between AIS A/B and AIS C/D in tetraplegics and paraplegics patients using a 2-sample *t*-test (1-tailed, unequal variances, α = 0.05).

The correlation between lesion level and spinal cord MRI (using the mean value along the levels C2 to C4) measures was assessed using an ordinal logit regression, entering an indicator of the lesion level, defined on the basis of the neurological lesion level, as response variable. Furthermore, multiple linear regressions were used to assess the correlation between qMRI parameters (using the mean value along the levels C2 to C4) and sensorimotor outcome measures (light touch, pin-prick, and SCIM scores). In order to determine if these associations were different in tSCI and pSCI, subject type indicator and type*predictor interaction terms were added; however, there was no evidence that associations were different in tSCI and pSCI and therefore the combined group was used in these analyses.

MT maps in PAM50 space were additionally analysed using general linear models (GML). Uncorrected voxel threshold of *p* = 0.001 was initially considered, and to account for multiple comparisons, family wise error correction (FWE) based on Gaussian random field theory, was applied. Only clusters surviving a corrected cluster threshold of *p* = 0.05 were reported (Friston et al., 1994). ANOVA tests were used in each voxel of interest to investigate spinal cord changes in SCI patients compared to healthy controls; and tSCI compared to pSCI. Additionally, associations between myelin sensitive microstructural changes and functional recovery (LEMS, light-touch, pin prick and SCIM scores) were investigated. All Statistical tests were corrected for age as a cofactor of no interest and only results with a significant threshold of *p* < 0.05 were reported.

### 2.7 Data availability

As the data are patient sensitive, the data will be made only available on request and fully anonymized.

## 3 Results

### 3.1 Demographic, clinical, and radiologic characteristics

There was no significant differences in age (ANOVA, *f* = 2.08 *p* = 0.135) or in sex (chi-square, *p* = 0.435) across the three groups (controls, tSCI and pSCI). tSCI patients were scanned on average 2.5 ± SD 3.9 years following the injury and were classified according to the American Spinal Injury Association Impairment Scale (AIS) as 6 AIS A, 2 AIS B, 1 AIS C and 6 AIS D. pSCI patients were scanned on average 3.6 ± 6.8 years following the injury and were classified as 8 AIS A, 4 AIS B, 2 AIS D and 1 AIS E.

### 3.2 Neurodegenerative gradient

**Fig. 2:**
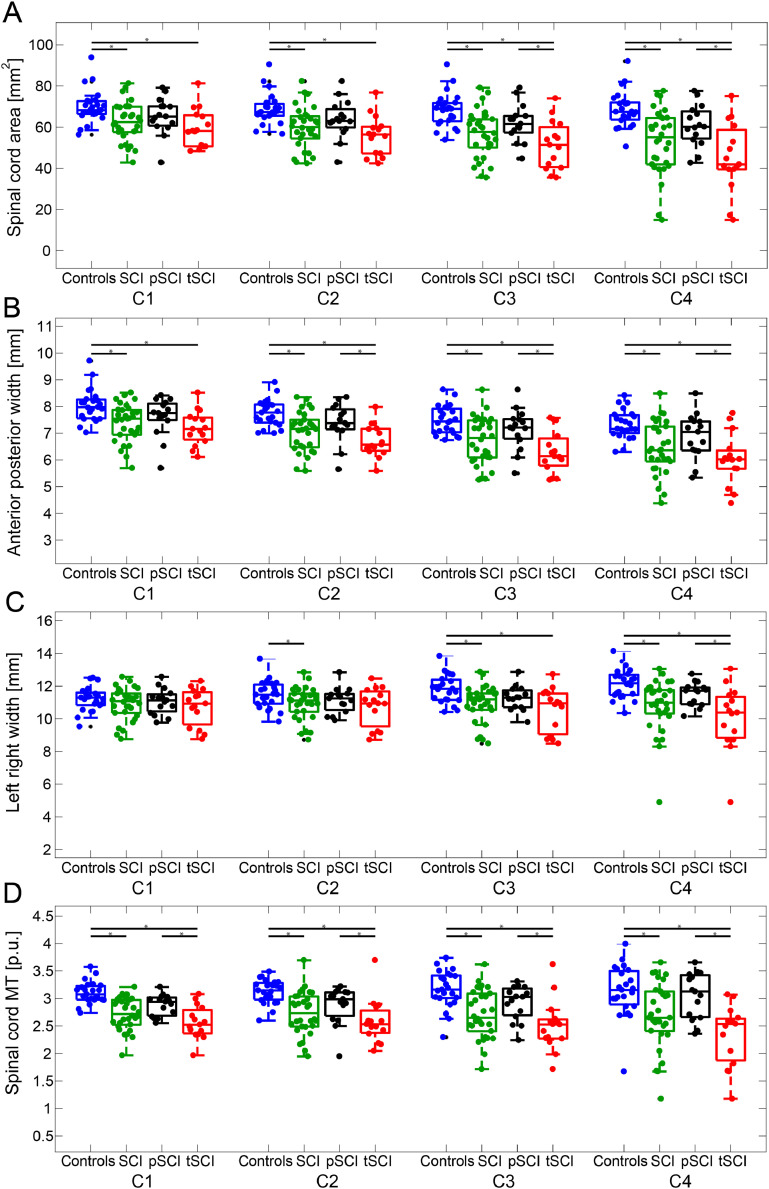
Spinal cord parameters from level C1 to C4. Morphometric and myelin-sensitive magnetization transfer parameters (coloured dots) at each individual cervical cord level (from C1 to C4) are shown (controls (blue), SCI (tSCI + pSCI, green), paraplegics (pSCI, black) and tetraplegics (tSCI, red)). Spinal cord parameters include: cross-section spinal cord area in A, its anterior posterior width (APW) in B, its left right width (LRW) in C and mean cord MT in D. Boxplots show the median (central mark), the 25th and 75th percentiles (bottom and top edges, respectively). The * symbol indicates significant differences (p < 0.05, family-wise corrected) between the connected groups. (For interpretation of the references to color in this figure legend, the reader is referred to the web version of this article.)

Cross-sectional cord area and its APW were decreased across all cervical cord levels in SCI patients (tSCI+pSCI) when compared to healthy controls (*p* < 0.05), while LRW showed decreases at levels C2, C3 and C4 (*p* < 0.05) (Fig. 2A–C, table 2). The magnitude of spinal cord area and shape changes in all SCI patients (tSCI+pSCI) was greater at lower cervical levels (i.e. closer to the lesion level, C4 > C3 > C2 > C1), defining a neurodegenerative gradient. Cord area decreased by 2.67 mm^2^ per cervical cord level in the caudal direction [95% Confidence Interval (CI) −4.48 to −0.86] (*p* = 0.004) and LRW decreased by 0.35 mm [95% CI −0.57 to −0.13] (*p* = 0.002) APW presented a negative gradient in caudal direction in all SCI patients (tSCI+pSCI) (APW decreased by 0.31 mm per cord level [95% CI −0.33 to −0.16] (*p* < 0.001)) and healthy controls (APW decreased by 0.24 mm per cord level [95% CI −0.39 to −0.24] (*p* < 0.001)), without a significant difference in the rate of change of the gradient (*p* = 0.197).

**Table 2:** MRI measures per cervical level in controls and spinal cord injury patients

MRI Parameters	Healthy controls mean ± sdt	SCI patients (pSCI+tSCI) mean ± sdt	Group differences [%]	p-value	[95% Confidence Interval]
cord area [mm^2^]					
c1	70.01 ± 8.15	62.44 ± 9.48	10.80	0.003	−12.49 to −2.65
c2	68.88 ± 7.59	59.79 ± 10.19	13.19	<0.001	−14.13 to −4.05
c3	68.42 ± 8.37	56.61 ± 11.64	17.26	<0.001	−17.47 to −6.13
c4	68.58 ± 8.77	53.02 ± 15.76	22.69	<0.001	−22.25 to −8.88
APW [mm]					
c1	8.04 ± 0.61	7.40 ± 0.71	7.96	<0.001	−0.99 to −0.29
c2	7.74 ± 0.50	7.06 ± 0.74	8.75	<0.001	−1.04 to −0.33
c3	7.49 ± 0.53	6.72 ± 0.88	10.28	<0.001	−1.16 to −0.39
c4	7.32 ± 0.54	6.46 ± 0.99	11.75	<0.001	−1.30 to −0.42
LRW [mm]					
c1	11.21 ± 0.74	10.89 ± 0.98	2.85	0.22	−0.83 to 0.19
c2	11.48 ± 0.85	10.93 ± 1.00	4.79	0.03	−1.05 to −0.05
c3	11.82 ± 0.85	10.91 ± 1.12	7.70	<0.001	−1.47 to −0.35
c4	12.15 ± 0.90	10.78 ± 1.63	11.28	<0.001	−2.06 to −0.69
Mean MT					
c1	3.10 ± 0.20	2.72 ± 0.29	12.26	<0.001	−0.54 to −0.23
c2	3.13 ± 0.22	2.73 ± 0.39	12.78	<0.001	−0.57 to −0.22
c3	3.17 ± 0.34	2.71 ± 0.44	14.51	<0.001	−0.68 to −0.23
c4	3.14 ± 0.47	2.69 ± 0.60	14.33	<0.001	−0.72 to −0.17

APW: anterior posterior width; LRW: left right width; MT: magnetization transfer.

**Fig. 3:**
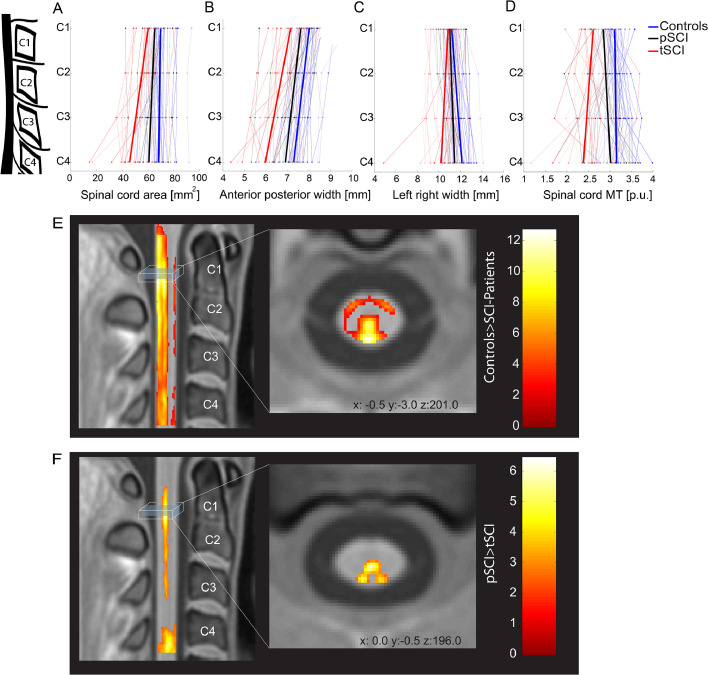
Neurodegeneration along the spinal cord. Spinal cord area (A), anterior posterior width (B), left right width (C) and myelin-sensitive magnetization transfer MT (D) parameters (coloured dots) along the cervical cord (from C1 to C4) in controls (blue), paraplegics (pSCI, black) and tetraplegics (tSCI, red). The light-coloured lines are connecting subject specific data; bold lines represent changes along the cervical cord axis for each group. Changes in myelin-sensitive magnetization transfer (MT) from the voxel-wise analysis in template space (PAM50(De Leener et al., 2018)) are shown in E and F. Overlay of statistical parametric maps (uncorrected p < 0.01, for illustrative purposes only) shows decreased myelin-sensitive MT in SCI patients compared to controls (E); and tetraplegic compared to paraplegic patients (F). The colour bar indicates the t score. (For interpretation of the references to color in this figure legend, the reader is referred to the web version of this article.)

In a within patient analysis (tSCI vs. pSCI), cord area decreased by 3.28 mm^2^ per cord level more in tSCI patients than in pSCI in the caudal direction, p = 0.011, [95% CI −5.99 to −0.57] (Fig. 3A). In tSCI patients, the APW showed a trend decrease when compared to pSCI patients (tSCI decreased by 0.15 mm more than in pSCI in the caudal direction, p = 0.07 [95% CI −0.31 to 0.0084]) (Fig. 3B); whereas LRW decreased by 0.36 mm more in tSCI than in pSCI in the caudal direction, p = 0.03 [95% CI −0.70 to −0.03]) (Fig. 3C). Comparing MRI metrics of AIS A/B vs AIS C/D in tSCI patients vs. pSCI patients across all spinal cord levels did not show significant differences (*p* > 0.05).

### 3.3 Pathophysiological changes

In SCI patients (tSCI+pSCI), myelin-sensitive MT was decreased across all cervical levels (z-score = 5.85, *x* = 3.5, *y* = 2.0, *z* = 195.5, *p* < 0.001, cluster extent (CE) = 2752) when compared to healthy controls (Figs. 2D, 3E; Table 2).

In a within patient group analysis (tSCI vs. pSCI), the decrease in myelin-sensitive MT in the cord was greater in tSCI patients at level C1 (z-score = 5.04, *x* = 0, *y* = −0.5, *z* = 195, *p* < 0.001, CE = 1036) and C4 (z-score = 5.41, *x* = −0.5, *y* = −3, *z* = 201, *p* < 0.001, CE = 2621) (Fig. 3F) when compared to pSCI. Mean MT in the cord decreased by 0.13% per cord level in tSCI patients when compared to pSCI in the caudal direction, *p* = 0.04 [95% CI−0.26 to −0.004] (Fig. 3D).

### 3.4 Associations between structural changes, lesion level and clinical outcome

**Fig. 4:**
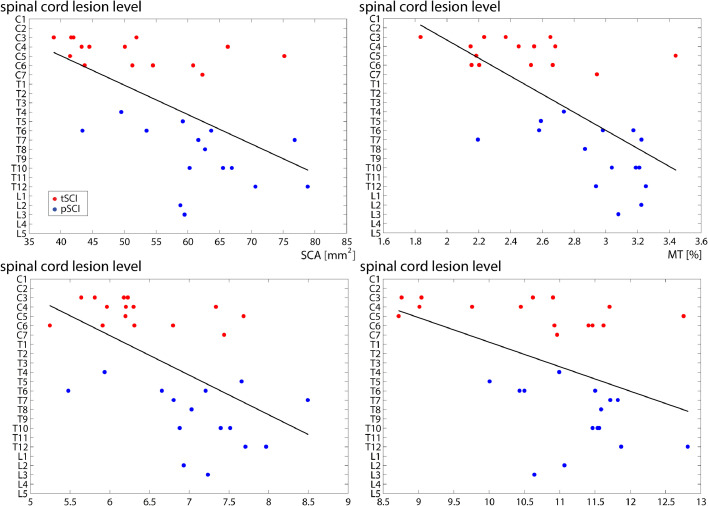
Correlations between lesion level and spinal cord parameters. On the y-axis is represented the lesion level which was defined on the basis of the neurological lesion level; on the x-axis the mean value of the individual MRI indices over cervical levels (from C1 to C4) in tSCI (red) and pSCI (blue) patients. (For interpretation of the references to color in this figure legend, the reader is referred to the web version of this article.)

In SCI patients (tSCI+pSCI), lesion level was associated with smaller mean cross-sectional cord area (*p* < 0.001, *r*^2^ = 0.146, [95% CI: 0.081−0.243]), mean APW (*p* < 0.001, *r*^2^ = 0.134; [CI: 1.011−3.228]), mean LRW (*p* = 0.003, *r*^2^ = 0.085; [CI: 0.369−1.792]), and mean cord MT (*p* < 0.001, *r*^2^ = 0.139; [CI: 1.923−6.083]) (Fig. 4).

**Fig. 5:**
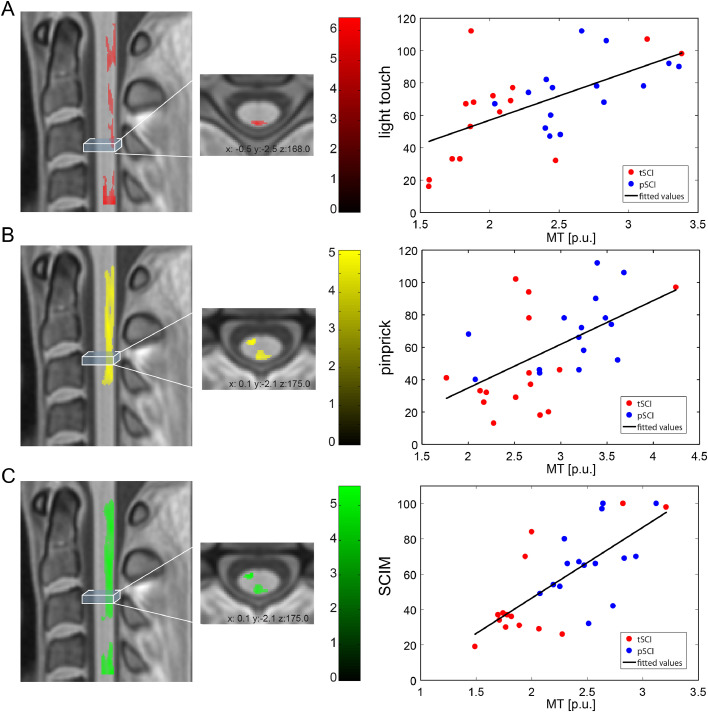
Correlations between clinical measures and spinal cord parameters. Correlations between myelin-sensitive magnetization transfer (MT) and light touch score (A); pin prick score (B), and SCIM score (C). Overlay of statistical parametric maps (uncorrected p < 0.01, for illustrative purposes only) on the left side (the colour bar indicates the t-score); correlation with extracted mean values from the significant clusters in the right column (for illustrative purposes only). (For interpretation of the references to color in this figure legend, the reader is referred to the web version of this article.)

In SCI patients (tSCI+pSCI), better pin prick score was associated with greater mean cord area (*p* = 0.016, *r*^2^ = 0.416; [CI: 0.201−1.826]) and APW (*p* = 0.007, *r*^2^ = 0.450; [CI: 4.869−27.646]). In SCI patients (tSCI+pSCI), higher light touch score was associated with greater mean APW (*p* =  0.033, *r*^2^ = 0.389; [CI: 1.069–23.248]) and higher SCIM score was associated with greater mean cord area (*p* = 0.041, *r*^2^ = 0.215; [CI: 0.042−1.805]) and mean APW (*p* = 0.035, *r*^2^ = 0.223; [CI: 1.041−25.902]). In SCI patients (tSCI+pSCI), greater mean MT in the dorsal column was associated with greater light touch score (z-score = 4.86, *x* = 2, *y* = −3, *z* = 143, *p* = 0.016, CE = 1166) (Fig. 5A). In SCI patients (tSCI+pSCI), greater mean MT in the dorsal columns (z-score = 5.07, *x* = −1, *y* = −3, *z* = 200.5, *p* < 0.001, CE = 3830) and spinothalamic tract (z-score = 4.85, *x* = −3, *y* = 2, *z* = 175, p = 0.024, CE = 1048) was associated with better pin prick score (Fig. 5B). In SCI patients (tSCI+pSCI), greater mean MT in the dorsal column (z-score = 4.39, *x* = −1, *y* = −2.5, *z* = 187, *p* < 0.001, CE = 3576) and spinothalamic tract (z-score = 4.06, *x* = −3, *y* = 2.5, *z* = 173, *p* = 0.045, CE = 839) was associated with better SCIM score (Fig. 5C). Spinal cord MRI indices were not associated with clinical scores of motor function (p > 0.05).

## 4 Discussion

This study shows a lesion level dependent neurodegenerative gradient across the cervical cord, with greater cord atrophy and myelin-sensitive MT reductions occurring in the proximity of the injury. These changes are less pronounced with increasing distance from the injury showing a gradient of neurodegeneration. In addition, changes in the spinal cord macro- and microstructure were associated with appropriate clinical outcomes. Tracking of a neurodegenerative gradient holds potential as a new neuroimaging biomarker to monitor treatment effects of regenerative and neuroprotective agents in interventional trials.

### 4.1 Characteristics of the neurodegenerative gradient

This is the first study to reveal a neurodegenerative gradient over several cervical segments (from C1 to C4) remote from a SCI in chronic tetraplegic and paraplegic patients. The extent of neurodegeneration was greatest in the proximity of the lesion and declining with increasing distance from the injury. Interestingly, only changes in the cords’ left-right direction showed a neurodegenerative gradient while the observed decrease in the anterior-posterior direction was equally decreased across all cervical levels. Changes in the cords’ LRW (Lundell, 2011) have been associated with retrograde degeneration of the corticospinal tract (Lemon, 2008); whereas changes in the APW (Grabher et al., 2015; Jutzeler et al., 2016; Lundell et al., 2011) have been associated with anterograde degeneration (i.e. Wallerian degeneration) in the posterior columns (Daniel and Strich, 1969). The lack of a neurodegenerative gradient within the dorsal columns – despite showing significant neurodegeneration – suggests that the magnitude of anterograde axonal degeneration in the posterior columns (i.e. Wallerian degeneration) (Daniel and Strich, 1969) is equally distributed across cervical levels (i.e. no gradient); while degeneration of the CST (captured by the LRW gradient) is greatest in the proximity of the injury epicentre and declines with increasing distance (Kalil and Schneider, 1975; Kerschensteiner et al., 2005). This gradient of neurodegeneration could be in part explained by the anatomical organization of the cord where an increasing number of axons enter or exit the cord from the cervical cord down to the thoracic cord. Therefore lesions at higher anatomical levels (rostrally) will injure more axons that project through it (more neurodegeneration) than an identical lesion more distant in the cord (caudally). In support of this point, higher lesion level has been directly associated with the magnitude of neurodegeneration (Jutzeler et al., 2016). However, the fact that significant neurodegenerative changes were detected in both tetraplegic and paraplegic patients in the high cervical cord independent of lesion severity suggests that a neurodegenerative gradient occurs above a SCI at any lesion level. It remains to be revealed whether a similar neurodegenerative gradient exists caudally to a SCI. This is of particular importance for repair studies which would aim to reduce the amount of degeneration in the caudal proximity of the injury via regenerating fibres. The fact that the lumbar cord undergoes neurodegenerative changes after a cervical SCI (David et al., 2019) speaks however for a neurodegenerative gradient occurring also below the level of injury.

### 4.2 Pathophysiological changes

By means of myelin-sensitive MT (Helms et al., 2008) we were able to disentangle one of the underlying pathophysiological mechanisms contributing to cord atrophy. Previous studies showed a centrifugal pattern of axonal degeneration starting from the lesion site (Bresnahan, 1978; Bresnahan et al., 1976; Kalil and Schneider, 1975; Kerschensteiner et al., 2005) with a gradual myelin loss propagation (Buss et al., 2005, 2004). This demyelination was shown to be a slow and continuous process covering more years after injury (Buss et al., 2005, 2004). Specifically, myelin proteins were still detectable 3 years after injury in the degenerating fibre tracts, long after the disappearance of the corresponding axons (Beirowski et al., 2005; Buss et al., 2004). As expected, the magnitude of the neurodegenerative gradient in the C1-C4 region was greater in tSCI patients when compared to pSCI, given the proximity of this investigated region to the site of injury. Therefore, myelin-sensitive MT, once proven in longitudinal studies, could be explored as a biomarker to monitor tract-specific myelin changes.

### 4.3 Associations between structural changes and clinical outcome

We found tract-specific associations with appropriate clinical outcomes. For instance, greater myelin-sensitive MT decreases in the dorsal column were directly linked to worse light touch scores, while a greater decrease of myelin-sensitive MT was associated with reduced pin prick and SCIM scores in both, dorsal column and spinothalamic tracts. In agreement with previous reports, greater changes in the cords’ APW were related to worse pin prick and light touch scores (Grabher et al., 2015; Lundell et al., 2011). The concurrent associations between sensory outcomes with atrophy in APW and functional independence measures with decreased myelin-sensitive MT in the ascending pathways, supports the notion that demyelination is a critical factor in the pathobiology of human SCI (Buss et al., 2005, 2004). Therefore, both APW and MT readouts hold promise as biomarkers for assessing changes of sensory function in traumatic SCI patients.

## 5 Limitations

This study has some limitations. Patients were on average 8 years older than healthy controls. However, all statistical analyses were corrected for the linear effect of age. MPM protocols (Weiskopf et al., 2013) provide maps sensitive to myelin (R1 and MT) and iron content (R2*). However, the quality of R1 and R2* maps at lower cervical cord levels was poor and could not be used for the analysis. Moreover, the interpretation of myelin-sensitive MT maps should be handled with caution as MT is an indirect marker for myelin and cannot directly describe underlying biological changes at the microscopic level. Nevertheless, histopathological studies have shown association between changes in MT and myelination (Schmierer et al., 2004; Turati et al., 2015).

## 6 Conclusion

In conclusion, this study shows that the magnitude of neurodegeneration decreases with evolving distance from the spinal injury along the cervical cord. In particular, the extent of neurodegeneration was more pronounced closer to the injury, was directly correlated to the level of the injury, and was associated with clinical impairments. Tracking remote cord pathology over several spinal segments is clinically eloquent. Future longitudinal study will have to test the potential of the MRI based neurodegenerative gradient measures whether these are sensitive to change over time and can differentiate between tSCI and pSCI patients. If proven, then MRI based neurodegenerative gradient measures could be used to monitor treatment effects of regenerative and neuroprotective agents.

## Disclosure statement

MSc Michela Azzarito reports no disclosures. Dr. Seif Maryam reports no disclosures. Dr. Sreenath Kyathanahally reports no disclosures. Prof. Armin Curt reports no disclosures. Prof. Patrick Freund reports no disclosures.

## Author contributions

Michela Azzarito: study concept and design; analysis and interpretation of data; writing the manuscript. Maryam Seif: critical revision of manuscript for intellectual content. Sreenath Kyathanahally: critical revision of manuscript for intellectual content. Armin Curt: critical revision of manuscript for intellectual content. Patrick Freund: study concept and design; critical revision of manuscript for intellectual content; study supervision.

## Study funding

This study is funded by ERA-NET NEURON (hMRIofSCI no: 32NE30_173678), the European Union's Horizon 2020 research and innovation program under the grant agreement No 681094, and the Swiss State Secretariat for Education, Research and Innovation (SERI) under contract number 15.0137, grants from Wings for life charity (INSPIRED) (No WFL-CH-007/14), and Eccellenza fellowship/181362 by SNSF. Open access of this publication is supported by the Wellcome Trust (091593/Z/10/Z).

## Declaration of Competing Interest

We declare no conflicts of interest.
